# Digital self-presentation inauthenticity and psychological distress among youth: a combined serial and parallel mediation model through online social comparison, digital identity confusion, and fear of missing out

**DOI:** 10.3389/fpsyg.2026.1892692

**Published:** 2026-10-01

**Authors:** Nie Ni, Aini Azeqa Ma’rof, Mohamad Naqiuddin Dahamat Azam, Zeinab Zaremohzzabieh, Seyedali Ahrari

**Affiliations:** 1 Department of Educational Science, Guangxi Minzu Normal University, Guangxi, Chongzuo, China; 2 Institute for Social Science Studies, Universiti Putra Malaysia, Serdang, Selangor, Malaysia; 3 Faculty of Human Ecology, Universiti Putra Malaysia, Serdang, Selangor, Malaysia; 4 Women and Family Studies Research Center, University of Religions and Denominations, Qom, Iran

**Keywords:** digital identity, fear of missing out, mediation analysis, psychological distress, self-presentation, social media

## Abstract

**Background:**

Modern youth navigate a complex duality between their physical selves and curated digital personas. This study investigated the relationship between digital self-presentation inauthenticity (DSPI) and psychological distress (PD) among Malaysian youth. Integrating the dramaturgical framework, self-discrepancy theory, and social comparison theory, the research tested three primary pathways: a serial chain mediated by online social comparison (OSC) and digital identity confusion (DIC), and two parallel pathways mediated independently by OSC and fear of missing out (FoMO).

**Methods:**

A quantitative study was conducted with data collected from 689 Malaysian youth. Hypotheses were tested using a combined serial and parallel mediation model via bootstrapped mediation analysis.

**Results:**

All three indirect effects were statistically significant. The serial mediation (inauthenticity → OSC → DIC → distress) was the most robust pathway (*b* = 0.078, 95% CI [0.047, 0.116]), followed by parallel mediation via FoMO (*b* = 0.094, 95% CI [0.059, 0.135]) and OSC (*b* = 0.049, 95% CI [0.018, 0.085]). Notably, the direct effect of inauthenticity on PD became non-significant after controlling for mediators, indicating that the direct association was no longer statistically significant after accounting for the proposed mediators. This pattern is consistent with indirect associations through these variables but, given the cross-sectional design, should not be interpreted as evidence of full mediation. The final model explained 33.1% of the variance in PD.

**Conclusion:**

The findings identify DIC and FoMO as critical predictors of youth mental health. This study provides theoretical insights into the mechanisms of digital identity and offers preliminary directions for future research and intervention development rather than empirically supported recommendations for clinical practice.

## 1 Introduction

The contemporary digital landscape has necessitated a dual existence for young people, characterized by an increasing discrepancy between the lived self and the curated online persona. While the strategic selection of esthetic content and the omission of unflattering realities have become normative digital behaviors, these actions constitute a systematic divergence between the “actual self” and the “presented self” (Degen, 2025). Although previous research has broadly linked social media (SM) use to diminished wellbeing (e.g., Hancock et al., 2022), the specific psychological mechanisms through which inauthentic self-presentation translates into pathology remain under-theorized. Thus, it is important to examine the psychological consequences of digital self-presentation inauthenticity (DSPI) and the mechanistic pathways specifically online social comparison (OSC), digital identity confusion (DIC), and fear of missing out (FoMO) that facilitate psychological distress (PD).

DSPI needs to be distinguished from other forms of online self-presentation with which it is often conflated. Impression management (Goffman, 1959) refers to the strategic regulation of information to influence others’ impressions and does not necessarily involve a perceived discrepancy between the presented and authentic self. Selective self-presentation involves choosing which aspects of the self to disclose or conceal, but such choices may remain subjectively authentic. Idealized self-presentation emphasizes desirable or aspirational characteristics, yet individuals may present an idealized self without experiencing it as inauthentic. Self-Discrepancy theory (S-DT; Higgins, 1987), in contrast, addresses broader discrepancies among actual, ideal, and ought selves but does not specifically locate these discrepancies within digital environments or focus on the subjective experience of maintaining an inauthentic online persona. DSPI, by contrast, specifically concerns an individual’s perceived mismatch between the self-constructed and maintained in digital spaces and their authentic sense of self (Gil-Or et al., 2015; Grieve et al., 2020; Michikyan, 2020). This distinction focuses on the person’s subjective experience of inauthenticity rather than simply the content, strategy, or desirability of an online presentation. Psychometric evidence supports this distinction: the social media Self-Presentation Discrepancy Scale (SMSDS) developed by Kusci and Bakır (2026) identified a three-factor structure comprising Reality Distortion and Exaggeration, Idealized Self-Presentation, and Strategic Impression Management, indicating that while strategic and idealized presentation are components of the broader phenomenon, DSPI specifically captures the perceived misalignment between presented and actual selves.

This focus on DSPI responds directly to a broader limitation in the existing literature. The “screen time” debate has generated a vast body of literature, yet it remains fundamentally inconclusive due to conflicting findings regarding psychological outcomes (Valkenburg et al., 2022). A critical limitation of existing studies is the treatment of SM use as a monolith, often focusing on the quantity of usage rather than the qualitative nature of digital engagement. By pivoting from usage frequency to the inauthenticity of the self-presented online, this study responds to calls for more nuanced, person-centered approaches in media effects research. Furthermore, the digital psychology literature remains disproportionately focused on Western populations, leaving a critical gap in our understanding of hyper-connected non-Western contexts. Malaysian youth represent a salient demographic for study, with SM penetration rates exceeding 88% in a population of 34 million (DataReportal, 2024). Investigating this population provides a unique cultural lens through which to test the universality of identity-related media effects.

Building on this rationale, the present study integrates major theoretical frameworks to map the trajectory from digital self-presentation to PD. Goffman’s (1959) dramaturgical framework provides the basis for understanding SM as a “front stage” where individuals strategically manage impressions. The psychological tension inherent in this management is captured by Higgins’s (1987) S-DT, which posits that the gap between the actual self and the idealized digital self generates dejection and anxiety. Furthermore, Festinger’s (1954) Social Comparison Theory (SCT) explains how curated self-presentation fosters an environment rife with upward comparisons that diminish self-evaluation. These processes are further contextualized by Valkenburg et al.’s (2022) differential susceptibility to media effects model, which accounts for individual differences in media influence, and Soh et al.’s (2024) affordance-driven digital identity processes framework, which elucidates how platform-specific features such as visibility and persistence constrain identity development.

Building on these frameworks, the present study generates a specific, testable mediation model in which DSPI predicts PD through three distinct pathways operating in parallel and in series. The primary serial chain runs from inauthenticity through online social comparison (OSC)—amplified by the comparison-rich environment of curated feeds—to digital identity confusion (DIC), representing the cognitive consequence of losing track of one’s authentic self. Simultaneously, two parallel pathways are hypothesized: one in which social comparison directly predicts distress, and another in which inauthentic self-presentation predicts FoMO, which in turn serves as a unique driver of PD. By testing these pathways concurrently, this research aims to move beyond simple correlations to provide a robust, mechanistic account of how inauthentic digital self-presentation harms young people, thereby identifying critical targets for psychological intervention in the digital age.

## 2 Theoretical framework and hypotheses development

The proposed model integrates Goffman’s (1959) dramaturgical framework, S-DT (Higgins, 1987), and SCT (Festinger, 1954) to examine how DSPI is statistically associated with PD through OSC, DIC, and FoMO. These three theories converge on a common premise: individuals are motivated to manage how they are perceived, to reconcile gaps between who they are and who they wish to be, and to evaluate themselves against others—processes that are amplified, accelerated, and rendered continuously visible in digital environments. From a dramaturgical perspective (Goffman, 1959), social-media profiles function as “front stage” performances in which users strategically select photos, captions, and posting times to project a desired impression, much like an actor managing a role before an audience. For instance, a user who repeatedly edits captions to sound more accomplished, or who posts about a social event only after confirming others attended, is engaging in impression management that may diverge from her backstage reality. S-DT (Higgins, 1987) explains why such divergence is psychologically costly: when an individual’s “actual self” (e.g., feeling lonely on a Friday night) is repeatedly juxtaposed with an “ideal self” projected online (e.g., posting a glamorous night-out photo), the resulting discrepancy generates dejection-related emotions such as sadness, anxiety, and dissatisfaction, as illustrated by the user who posts about a promotion while privately struggling with impostor feelings. SCT (Festinger, 1954) accounts for the evaluative dimension of this process: because social media offers no objective standard for attractiveness, success, or popularity, users rely on comparison with others particularly upward comparison with seemingly more successful, attractive, or socially connected peers such that a student who compares her modest internship to a classmate’s viral LinkedIn post about a prestigious placement may experience diminished self-evaluation, even if her own achievement is objectively commendable. These theoretical lenses illuminate a common digital sequence: a young adult posts a heavily filtered vacation photo on Instagram (dramaturgical performance), privately feels that the polished image misrepresents her actual experience (self-discrepancy), and then immediately scrolls through peers’ equally curated posts of career achievements and romantic milestones (social comparison).

Research on social-media self-expression further indicates that users may experience a tension between authentic and idealized self-presentation, with greater authenticity being associated with better wellbeing (Bailey et al., 2020). Wong and Hamza (2025) found across three waves (*N* = 1,217) that false-deceptive self-presentation and comparison/impression-management self-presentation predicted lower self-concept clarity and greater depressive symptoms, with random-intercept cross-lagged panel models confirming bidirectional within-person associations. Similarly, Nor et al. (2025) demonstrated that false self-presentation significantly elevated fear of negative evaluation, which in turn drove excessive social-media use, while social comparison operated through validation-seeking mechanisms. These findings empirically corroborate the theoretical sequence outlined above: the Instagram user who filters her vacation photo (Goffman), feels the gap between her presented and actual self (Higgins), and then compares her experience to peers’ curated feeds (Festinger) is not merely engaging in isolated digital behaviors but is traversing a theoretically specified pathway that recent longitudinal and large-scale evidence links to lower self-concept clarity, greater fear of negative evaluation, and heightened depressive symptoms. These perspectives provide a theoretical basis for the proposed direct associations and indirect pathways.

### 2.1 Digital self-presentation inauthenticity and online social comparison

Social media allows individuals to construct their own self-presentations while simultaneously viewing and evaluating those of others. From a dramaturgical perspective, users function as both performers and audiences (Goffman, 1959). Research distinguishing authentic from idealized online self-expression suggests that the manner in which individuals present themselves digitally may be relevant to psychological functioning (Bailey et al., 2020). When individuals perceive their digital self-presentation as inconsistent with their authentic selves, they may become more attentive to how their presentation compares with those of other users. Social-media environments are particularly relevant because they combine visual self-presentation, continuous social evaluation, and quantified feedback such as likes, comments, and views. Platforms such as Instagram and TikTok also expose users to selectively curated representations of appearance, achievements, relationships, and lifestyles. Experimental evidence supports the relevance of these features. Di Gesto et al. (2023) found that exposure to Instagram posts with high numbers of likes increased body dissatisfaction and social physique anxiety among young women, while disclaimers concerning digital image enhancement moderated these responses. These findings illustrate how quantified social endorsement and idealized visual content can shape self-evaluation and affective responses.

SCT proposes that individuals evaluate themselves relative to others, particularly when objective standards are unavailable (Festinger, 1954). Social media provides abundant comparison targets that are often selectively constructed and socially desirable. A recent systematic review and meta-analysis likewise found that greater online social comparison was associated with greater body-image concerns across 83 studies (Bonfanti et al., 2025), supporting the relevance of comparative processes within visually oriented social-media environments. Furthermore, Zhao and Li (2026) found across a 12-week longitudinal study (*N* = 398) that upward digital social comparison exerted sustained negative effects on self-concept through the mechanisms of self-discrepancy and ideal internalization, with platform modality (particularly visual platforms) moderating these effects. For individuals uncertain about the authenticity of their own online presentation, these curated representations may become salient standards for evaluating their identity and social position. Türk-Kurtça and Doğru (2026) found in a structural equation model (*N* = 440) that digital esthetics influenced social-media self-presentation through the serial mediation of social-media comparison and false self-presentation (*β* = 0.59, *p* < 0.001; *β* = 0.37, *p* < 0.001), with good model fit (CFI = 0.96, RMSEA = 0.05). Existing research links self-presentation and social comparison, although the specific association between perceived DSPI and OSC remains underexamined (Yang et al., 2018; Aryal et al., 2025). Because the present study is correlational, the experimental findings of Di Gesto et al. (2023) are used to strengthen the theoretical rationale rather than establish causality. Therefore, it was hypothesized that:

*H1*. Digital self-presentation inauthenticity is positively associated with online social comparison.

### 2.2 Online social comparison and digital identity confusion

OSC may extend beyond comparative self-evaluation to broader perceptions of identity. Social media exposes individuals to numerous standards concerning appearance, achievement, relationships, lifestyles, and social status. Yang et al. (2018) found that social-media comparison of ability was associated with lower identity clarity, while a separate study linked social-media comparison with rumination and subsequent identity distress among college students (Yang et al., 2018). Repeated comparison may draw attention to discrepancies between how individuals understand themselves and how they believe they should present themselves online, potentially making it more difficult to maintain a coherent identity across contexts. From a self-discrepancy perspective, persistent awareness of differences among self-representations may be psychologically consequential (Higgins, 1987). Empirical evidence supports an association between social-media comparison and identity-related difficulties. Yang et al. (2018) found that social-media comparison of ability was associated with rumination, which was subsequently related to identity distress among college students. Although identity distress is not synonymous with DIC, the finding suggests that comparative processes may extend to broader identity experiences. Research on self-concept clarity likewise indicates that coherence and stability in self-beliefs are associated with psychological functioning (Campbell et al., 1996). Zhao and Li (2026) further supports this pathway by demonstrating that self-discrepancy and ideal internalization function as key mediators linking digital social comparison to impaired self-concept over time. Additionally, Türk-Kurtça and Doğru (2026) found in a sample of 440 participants that appearance-related social-media consciousness significantly predicted false self-presentation, which in turn was associated with social-media fatigue, underscoring the role of identity-related processes in digital contexts. Accordingly, evidence that social-media comparison is related to identity clarity and identity distress provides empirical support for considering OSC as a correlate of identity-related uncertainty, while the present study specifically examines DIC. Accordingly, persistent OSC may be associated with greater difficulty maintaining a coherent sense of identity across digital and offline contexts. Therefore, it was hypothesized that:

*H2*. Online social comparison is positively associated with digital identity confusion.

### 2.3 Digital self-presentation inauthenticity and fear of missing out

FoMO represents a distinct response to digitally mediated social information. It refers to a pervasive concern that others are experiencing rewarding events in one’s absence and a desire to remain connected with those experiences (Przybylski et al., 2013). Social media may intensify FoMO by providing continuous access to information about others’ activities, relationships, achievements, and social experiences. The highly visible and socially evaluative nature of online self-presentation may make socially desirable experiences particularly salient, providing a context in which concerns about one’s own participation and social connectedness become relevant. DSPI may be relevant to FoMO because perceived discrepancies between the authentic and digitally presented selves may increase attention to socially desirable experiences displayed by others. From a dramaturgical perspective, individuals managing their own digital performances are simultaneously exposed to the performances of others (Goffman, 1959). This ongoing exposure may heighten concern about whether one’s own experiences are sufficiently socially rewarding or whether important experiences are occurring without one’s participation. Ho et al. (2026) found in a large-scale study (*N* = 1,534 university students) that FoMO was significantly associated with psychological distress through the mediating mechanism of Facebook addiction, with 91% of the sample reporting psychological distress. Cahyaningthyas and Huwae (2026) similarly reported a significant positive correlation between FoMO and psychological distress (*r* = 0.20, *p* < 0.01) among 298 millennials. Given the established association of FoMO with social-media engagement and poorer psychological outcomes (Przybylski et al., 2013), FoMO represents a theoretically distinct social-exclusion-related correlate of psychological functioning. Therefore, it was hypothesized that:

*H3*. Digital self-presentation inauthenticity is positively associated with fear of missing out.

### 2.4 Digital identity confusion and psychological distress

DIC concerns uncertainty, inconsistency, or reduced coherence in how individuals understand themselves across digital and offline contexts. Such experiences may be particularly relevant during adolescence and emerging adulthood, when identity development remains important. Although identity exploration is normative, persistent uncertainty about the coherence or continuity of the self may be psychologically burdensome. S-DT proposes that discrepancies among self-representations may be associated with negative emotional experiences (Higgins, 1987). Consistent with this perspective, Michikyan (2020) found that identity coherence and identity confusion were associated with different forms of online self-presentation among emerging adults, with greater identity confusion linked to less truthful and more socially desirable self-presentation. In digital environments, individuals may maintain multiple self-presentations across audiences while attempting to preserve a coherent identity. When these representations are experienced as inconsistent, uncertainty regarding the authenticity or stability of the self may be associated with psychological difficulties. Research on self-concept clarity similarly links coherence and stability of self-beliefs with psychological functioning (Campbell et al., 1996). Digital affordances such as editability, persistence, and audience evaluation may further shape these experiences (Shulman, 2022; Soh et al., 2024). Wong and Hamza (2025) provided longitudinal evidence that false-deceptive self-presentation and depressive symptoms are reciprocally related across time at the within-person level, suggesting that identity-related digital behaviors and psychological distress may mutually reinforce each other. These findings support the relevance of identity coherence and confusion to understanding psychological adjustment in digitally mediated contexts. Therefore, it was hypothesized that:

*H4*. Digital identity confusion is positively associated with psychological distress.

### 2.5 Fear of missing out and psychological distress

FoMO provides a second, conceptually distinct pathway to PD. Individuals experiencing FoMO may have persistent concerns about social exclusion, disconnection, and inadequate participation (Przybylski et al., 2013). Continuous access to social information may reinforce these concerns by repeatedly exposing individuals to experiences from which they are absent. Although monitoring social-media content may reduce uncertainty temporarily, it may also provide further opportunities to perceive exclusion or missed experiences. FoMO is distinct from DIC: DIC concerns uncertainty about the coherence and continuity of one’s identity, whereas FoMO concerns apprehension about exclusion from rewarding experiences. Ho et al. (2026) demonstrated that FoMO exerts its influence on psychological distress through behavioral pathways (i.e., Facebook addiction), highlighting the distinct affective-behavioral mechanism underlying this pathway. The large-scale evidence from 1,534 participants with a 91% prevalence of psychological distress underscores the practical significance of FoMO as a correlate of distress in emerging-adult populations. Evidence linking FoMO with poorer mood, lower life satisfaction, and greater social-media engagement supports its relevance to psychological wellbeing (Przybylski et al., 2013). Therefore, it was hypothesized that:

*H5*. Fear of missing out is positively associated with psychological distress.

### 2.6 Serial indirect association through online social comparison and digital identity confusion

The preceding hypotheses provide the basis for a serial pathway from DSPI to PD through OSC and DIC. Perceived inauthenticity may be associated with greater attention to others’ digital presentations, which may correspond with more frequent comparison. Comparative evaluation may then be associated with greater uncertainty concerning identity coherence, which in turn may be associated with PD. This proposed sequence integrates the dramaturgical, social-comparison, and self-discrepancy perspectives. Türk-Kurtça and Doğru (2026) provided direct empirical support for this serial mechanism by demonstrating that social-media comparison and false self-presentation sequentially mediate the relationship between digital esthetics and social-media self-presentation outcomes. Zhao and Li (2026) further validated that self-discrepancy and ideal internalization mediate the longitudinal effects of digital social comparison on self-concept. Evidence that online social comparison is associated with identity distress and lower identity clarity (Yang et al., 2018) and that identity confusion is related to less truthful and more socially desirable online self-presentation (Michikyan, 2020) provides empirical support for examining these constructs within a broader statistical model. Although previous research has examined components of this sequence, fewer studies have integrated perceived DSPI, social comparison, and identity-related uncertainty within a single model. The present study therefore examines whether OSC and DIC statistically account for an indirect association between DSPI and PD. Because all variables were assessed concurrently, this ordering represents a theoretically specified statistical model rather than an assumed temporal or causal sequence. Therefore, it was hypothesized that:

*H6a*. Digital self-presentation inauthenticity has a positive indirect association with psychological distress through the serial pathway of online social comparison and digital identity confusion.

### 2.7 Specific indirect association through online social comparison

OSC may also represent a specific indirect pathway to PD that does not involve DIC. Social media provides numerous, highly curated comparison targets, potentially encouraging individuals to evaluate their ordinary experiences against selectively presented positive information about others (Festinger, 1954). The broader literature indicates that online social comparison is associated with adverse self-evaluative outcomes, including body-image concerns (Bonfanti et al., 2025). Zhao and Li (2026) provide direct evidence that digital social comparison exerts sustained negative effects on self-concept through self-discrepancy and ideal internalization, independent of broader identity-confusion processes. Individuals who perceive their digital self-presentation as inauthentic may be particularly attentive to such comparisons because of concerns about how their online identity corresponds with socially desirable standards. Comparative evaluation may be associated with dissatisfaction, negative self-evaluation, or concerns about social standing even without broader identity confusion. Thus, OSC may represent both a component of the proposed serial pathway and a distinct statistical pathway between DSPI and PD. Therefore, it was hypothesized that:

*H6b*. Digital self-presentation inauthenticity has a positive indirect association with psychological distress through online social comparison.

### 2.8 Specific indirect association through fear of missing out

FoMO provides a second specific indirect pathway. Whereas OSC concerns comparative self-evaluation, FoMO concerns apprehension that others are experiencing rewarding events without one’s participation (Przybylski et al., 2013). These processes may co-occur but remain conceptually distinct. Recent longitudinal work also suggests that online self-presentation and identity-related processes can be reciprocally related over time, highlighting the dynamic relationship between digital self-presentation and identity development (Noon et al., 2024). Ho et al. (2026) provided large-scale evidence (*N* = 1,534) that FoMO operates as a distinct pathway to psychological distress through behavioral mechanisms (Facebook addiction), independent of identity-related cognitive processes. Cahyaningthyas and Huwae (2026) further confirmed the direct association between FoMO and psychological distress (*r* = 0.20, *p* < 0.01) in a sample of 298 millennials. Perceived DSPI may be associated with greater sensitivity to the social experiences displayed by others, while continuous exposure to such experiences may correspond with concerns about exclusion and social disconnection. Testing FoMO alongside the OSC–DIC pathway allows the present study to examine whether social-exclusion concerns represent an additional statistical pathway between DSPI and PD. Accordingly, it was hypothesized that:

*H6c*. Digital self-presentation inauthenticity has a positive indirect association with psychological distress through fear of missing out.

Taken together, the framework positions DSPI as the predictor and PD as the outcome, with OSC, DIC, and FoMO representing theoretically distinguishable psychological processes. H1–H3 address the proposed associations among DSPI, OSC, DIC, and FoMO; H4–H5 address the associations of DIC and FoMO with PD; and H6a–H6c examine the proposed indirect associations between DSPI and PD. The direct DSPI–PD association is estimated within the mediation model but is not assigned a separate hypothesis because the theoretical focus is on the proposed indirect pathways. Because all variables were measured concurrently, the proposed pathways are interpreted as theoretically informed statistical associations rather than evidence of causal mediation.

## 3 Methods

### 3.1 Participants and design

The sample comprised 689 Malaysian youth (53.0% female, 43.0% male, 4.0% non-binary/other) recruited through a multi-stage cluster sampling procedure. In the first stage, seven Malaysian states were selected as clusters to represent the country’s major geographic regions: Peninsular Malaysia (Selangor, Johor, Penang, Kelantan) and East Malaysia (Sabah, Sarawak, Labuan). These states were chosen because they collectively capture the ethnic, socioeconomic, and urban–rural diversity of the Malaysian youth population. Within each selected state, participants were recruited through purposive online sampling and snowball referrals, both non-probability techniques, to ensure adequate representation of the target demographic across age, gender, ethnicity, and educational background. Participants were aged 15–30 years (*M* = 20.80, *SD* = 3.20). This range was selected to capture developmental processes relevant to emerging adulthood (Arnett and Mitra, 2020), including identity exploration and self-focus. Participants aged 15–17 were included given the high prevalence of social-media use among adolescents (DataReportal, 2024) and the relevance of this developmental period to digital identity formation (Erikson, 1968). Although the age range reflects developmental heterogeneity, 76.2% were aged 18–25 years, while 11.5% were aged 15–17 and 12.3% were aged 26–30.

The sample comprised Malay (55.0%), Chinese (25.0%), Indian (12.0%), and other indigenous groups (8.0%). These proportions closely mirror Malaysian national census estimates (57.0, 23.0, 12.0, and 8.0%, respectively; Department of Statistics Malaysia (2023). The cluster sampling design, in which states were selected to represent Malaysia’s major geographic and demographic regions, provides a methodological basis for the sample’s ethnic and geographic diversity. However, because the final stage of recruitment within each state used purposive and snowball sampling, the sample cannot be considered a fully probability-based national sample. The similarity with census proportions is therefore reported as evidence of the sample’s demographic breadth and as a strength of the cluster-based recruitment strategy, while acknowledging that population-level generalizability should be interpreted with caution. Regarding educational attainment, 51.2% held or were pursuing a Bachelor’s degree, 21.9% were pre-university students, 14.2% were enrolled in secondary school, and 12.8% were postgraduate students. Participants reported 5.30 h (*SD* = 1.80) of daily social-media use on average, with TikTok (31.2%) and Instagram (28.7%) as the primary platforms. Eligibility required Malaysian residency, age 15–30 years, and daily use of at least one social-media platform. An *a priori* power analysis indicated that the final sample of 689 exceeded the minimum sample of 460 required to detect small-to-medium indirect effects in complex mediation models, with power (1 − *β*) > 0.95 (Fritz and MacKinnon, 2007). The multi-stage cluster sampling procedure, combining geographic clustering with purposive online recruitment within states, was designed to enhance the demographic and geographic diversity of the sample. This approach captures the ethnic and regional heterogeneity of Malaysian youth more effectively than a single-site or purely convenience-based sample, while acknowledging that the non-probability final stage limits strict population-level inference.

### 3.2 Measures

#### 3.2.1 Digital self-presentation inauthenticity

DSPI was measured using a 6-item scale developed for this study, grounded in Higgins’s (1987) S-DT and Goffman’s (1959) dramaturgical theory. The items assess the perceived divergence between the user’s online persona and their actual self across domains such as appearance management and lifestyle portrayal (e.g., “The version of myself I present on social media is quite different from who I really am in person”). Participants responded on a 5-point Likert scale ranging from 1 (*strongly disagree*) to 5 (*strongly agree*). In the present sample, the scale demonstrated strong internal consistency. An initial pool of 18 items was developed to represent three domains: online–offline self-discrepancy, subjective awareness of inauthenticity, and sustained misrepresentation. Three researchers with expertise in digital psychology and identity evaluated the items for relevance, representativeness, clarity, and cultural appropriateness for Malaysian youth. Items with content validity index (CVI) values below 0.78 were revised or removed, resulting in 12 items. The revised scale was pilot tested with 45 Malaysian youth (*M*age = 21.30, *SD* = 2.90; 55.6% female), and six items were retained based on factor loadings, item-total correlations, and conceptual coverage. Pilot reliability was acceptable (*α* = 0.79). These procedures provided initial evidence of cultural suitability.

In the main sample, DSPI demonstrated good internal consistency (*α* = 0.82). Confirmatory factor analysis (CFA) supported a unidimensional structure (CFI = 0.91, RMSEA = 0.058, SRMR = 0.047), with standardized loadings of 0.51–0.79 (*p* < 0.001) and average variance extracted (AVE) = 0.48. Heterotrait–monotrait (HTMT) ratios with all other constructs were below 0.85 (range = 0.41–0.62), supporting discriminant validity. These findings provide initial evidence of reliability, factorial validity, convergent validity, and discriminant validity, although further validation in independent and more diverse samples is warranted.

#### 3.2.2 Online social comparison

Online social comparison (OSC) was assessed using a 5-item measure adapted from the Social Comparison Orientation Scale (Gibbons and Buunk, 1999). Five of the original 11 items were selected based on conceptual relevance to online comparison, factor loadings above 0.60 in previous validation research, and conceptual distinctiveness from the present constructs. Wording was modified to explicitly reference social-media contexts while preserving the original meaning. For example, an item referring to general social comparison was reworded as “Social media makes me feel like others are living more fulfilling lives than I am.” The scale was administered in English; no translation was undertaken because English is widely used in Malaysian tertiary education and has been used in previous Malaysian research involving social comparison (Kam and Prihadi, 2021). Three researchers reviewed the adapted items for content validity and cultural appropriateness, and the measure was pilot tested with the same 45 Malaysian youth (*α* = 0.76). In the main sample, OSC demonstrated acceptable reliability (*α* = 0.78). CFA supported a unidimensional structure (CFI = 0.93, RMSEA = 0.052, SRMR = 0.044), with loadings of 0.48–0.74 (*p* < 0.001) and AVE = 0.46. HTMT ratios with the other constructs ranged from 0.41 to 0.62, supporting discriminant validity. Previous Malaysian applications of social-comparison measures also support the contextual applicability of the construct (Ma’rof and Abdullah, 2024).

#### 3.2.3 Digital identity confusion

Digital identity confusion (DIC) was assessed using a 5-item measure developed from Soh et al.’s (2024) affordance-driven identity processes framework and Erikson’s (1968) identity theory. The measure captures uncertainty about the authentic self, fragmentation across platforms, difficulty integrating online and offline identities, and perceived self-concept destabilization (e.g., “I feel confused about who I really am because of the different identities I present online”). Responses ranged from 1 (strongly disagree) to 5 (strongly agree). An initial pool of 15 items was developed and reviewed by three experts in digital psychology and identity research for theoretical relevance, cultural appropriateness, and clarity for Malaysian youth. Items with CVI values below 0.78 were revised or removed, producing nine items. Pilot testing with 45 Malaysian youth resulted in five retained items based on factor loadings, item-total correlations, and conceptual coverage (*α* = 0.75). These procedures provided initial evidence of cultural suitability. In the main sample, DIC demonstrated good internal consistency (*α* = 0.78). CFA supported a unidimensional structure (CFI = 0.94, RMSEA = 0.049, SRMR = 0.042), with standardized loadings of 0.52–0.76 (*p* < 0.001) and AVE = 0.51. HTMT ratios ranged from 0.42 to 0.68 and remained below 0.85, supporting discriminant validity from DSPI, OSC, FoMO, and PD.

#### 3.2.4 Fear of missing out

Fear of missing out (FoMO) was assessed using a 4-item abbreviated version of the scale developed by Przybylski et al. (2013). Four of the original 10 items were selected based on factor loadings reported in previous validation studies, coverage of the two core FoMO dimensions (concern about missing rewarding experiences and the need to remain connected), and evidence of reliability in previous Malaysian research (Wong and McLellan, 2024; Tan et al., 2023). Wording was retained without translation because the scale was administered in English, consistent with previous Malaysian applications and the linguistic context of the tertiary-educated sample. A brief glossary and clarification were provided where necessary. Pilot testing with 45 Malaysian youth demonstrated acceptable reliability (*α* = 0.76). In the main sample, FoMO demonstrated acceptable internal consistency (*α* = 0.72). CFA indicated acceptable fit (CFI = 0.91, RMSEA = 0.057, SRMR = 0.048), with loadings of 0.45–0.70 (*p* < 0.001) and AVE = 0.44. Although AVE was slightly below 0.50, this was considered acceptable given the abbreviated four-item measure. HTMT ratios ranged from 0.39 to 0.57, supporting discriminant validity. Previous research has also reported acceptable psychometric properties of FoMO across populations, including Malaysian samples (Przybylski et al., 2013; Wong and McLellan, 2024; Tan et al., 2023).

#### 3.2.5 Psychological distress

Psychological distress (PD) was assessed using a 6-item composite measure developed for the present study to capture general psychological distress and distress in the context of digital engagement. The measure is not equivalent to the Kessler Psychological Distress Scale (Kessler et al., 1997), because it combines three K6-derived items with three digitally tailored items. Participants responded from 1 (never) to 5 (all the time). The three K6-derived items assess nervousness, hopelessness, and sadness, whereas the three digitally tailored items assess feelings of inadequacy during online comparison, anxiety related to managing multiple online identities, and emotional exhaustion from maintaining an online persona. The measure was designed to retain indicators of general distress while capturing affective distress relevant to digital engagement. Importantly, the digital items assess distress responses rather than the underlying processes represented by the mediators. Thus, the online-comparison item assesses feelings of inadequacy rather than the tendency to engage in OSC, while the identity- and persona-related items assess anxiety and exhaustion rather than DIC or DSPI themselves. All items were reviewed by experts for content validity and cultural appropriateness and pilot tested with 45 Malaysian youth (*α* = 0.79), providing preliminary evidence of cultural suitability. In the main sample, PD demonstrated strong internal consistency (*α* = 0.84). CFA supported a unidimensional structure (CFI = 0.92, RMSEA = 0.055, SRMR = 0.046), with standardized loadings of 0.48–0.79 (*p* < 0.001) and AVE = 0.53.

To assess factorial and discriminant validity, competing measurement models were compared. The hypothesized five-factor model showed substantially better fit than a single-factor model and alternative models collapsing pairs of constructs. All HTMT ratios among DSPI, OSC, DIC, FoMO, and PD were below 0.85, with the highest value being 0.68. These findings support the empirical distinctiveness of the five constructs, including PD from OSC despite the digitally contextualized comparison item. Because three PD items were developed specifically for the digital context, the measure should not be treated as interchangeable with the full K6, and comparisons with studies using the full K6 should be interpreted cautiously.

### 3.3 Procedure

Data were collected between February and April 2026 using a structured self-administered online questionnaire hosted on Google Forms. The study involved human participants and received ethical approval from Universiti Putra Malaysia Sabah (Ethics Approval No. JKEUPM-2025-1262). Participants provided electronic informed consent before completing the survey; for those younger than 18 years, electronic parental or legal guardian consent and participant assent were obtained. The survey took approximately 16 min to complete. Participation was voluntary and uncompensated, and responses were anonymized with no personally identifying information retained. All procedures complied with the ethical standards of Universiti Putra Malaysia Sabah, the Declaration of Helsinki, and the American Psychological Association (APA) Code of Ethics.

### 3.4 Data analysis

Data were analyzed using IBM SPSS Statistics 28 and PROCESS macro 4.2 (Hayes, 2018). Preliminary analyses included descriptive statistics, reliability analysis, Pearson correlations, multiple regression, and one-way ANOVA. Multiple regression examined the unique associations of DSPI, OSC, DIC, and FoMO with PD, controlling for age and gender; ANOVA examined PD differences across primary social media platforms. Indirect effects were tested using PROCESS Model 81 (Hayes, 2018), with DSPI as the predictor, PD as the outcome, and OSC, DIC, and FoMO as mediators. Model 81 estimates the direct DSPI–PD association, the indirect effects through OSC (a1 × b1), FoMO (a2 × b3), and the serial pathway DSPI → OSC → DIC → PD (a1 × d21 × b2), as well as all paths among the mediators, including the path from OSC to DIC (d21). All paths estimated in the model are reported in Figure 1 and Table 1. Age, gender, and education were included as covariates in the mediation model. Education was coded categorically (secondary, pre-university/college, university/postgraduate). The separate regression analysis included age and gender because education did not significantly contribute to PD; this analysis was conducted to estimate unique concurrent associations and standardized coefficients (*β*). Indirect effects were tested using 5,000 bootstrap resamples and 95% CIs; effects were significant when CIs excluded zero (Hayes, 2018).

**Figure 1:**
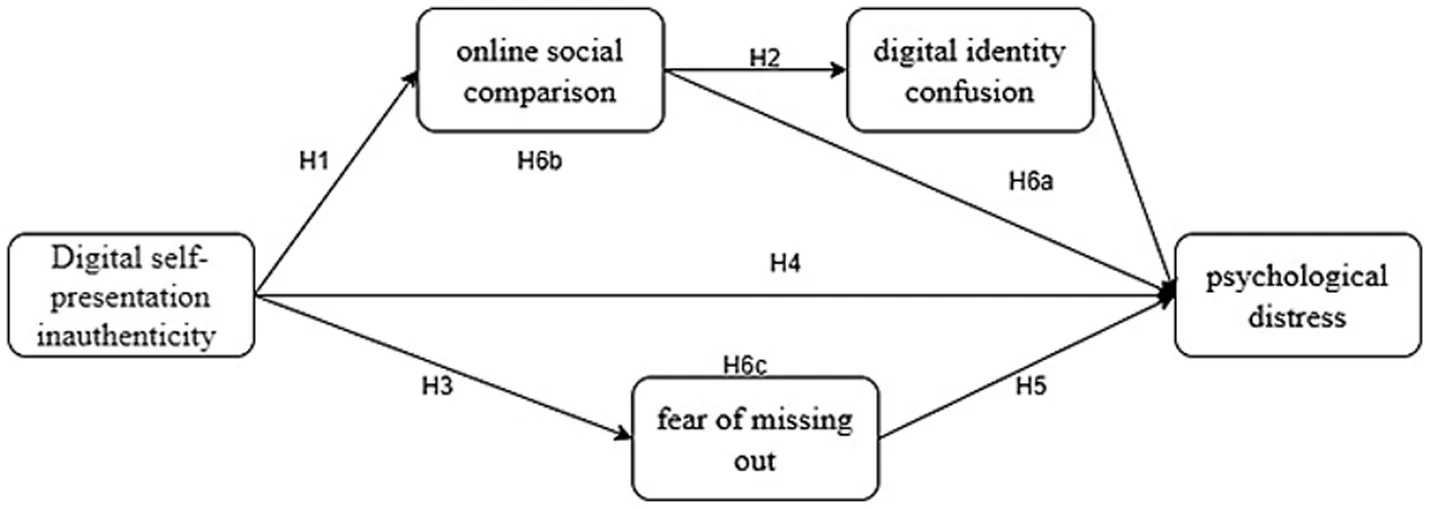
Conceptual framework of the relationships among study variables.

**Table 1:** Indirect effects of DSPI on psychological distress via OSC, DIC, and FoMO (*N* = 689).

Path	Effect (𝑏)	*SE*	LLCI	ULCI
Total indirect effect	0.222	0.029	0.168	0.283
H5a: DSPI → OSC → DIC → PD	0.078	0.018	0.047	0.116
H5b: DSPI → OSC → PD	0.049	0.020	0.018	0.085
H5c: DSPI → FoMO → PD	0.094	0.019	0.059	0.135

Bootstrap CI based on 5,000 resamples. CI excluding zero = significant indirect effect. DSPI, digital self-presentation inauthenticity; OSC, online social comparison; DIC, digital identity confusion; FoMO, fear of missing out; PD, psychological distress.

Sensitivity analyses examined age-related differences. Age was tested as a continuous moderator, with no significant interactions (all ps > 0.10). Participants were also grouped into 15–17 years (*n* = 79), 18–25 years (*n* = 525), and 26–30 years (*n* = 85). Multi-group structural equation modeling (SEM) showed no significant difference between unconstrained and constrained models, Δχ^2^ = 18.65, Δdf = 16, *p* = 0.287. Indirect effects were significant across groups with overlapping CIs. Thus, no significant age-group differences were observed, although the smaller groups warrant caution and do not establish developmental equivalence.

## 4 Results

### 4.1 Descriptive statistics and internal consistency

Descriptive statistics and internal consistency coefficients for all primary study variables are summarized in Table 2. On a 5-point Likert scale, mean scores for the observed constructs ranged from 2.91 to 3.43. Notably, PD exhibited the highest mean score (*M* = 3.43, *SD* = 0.56), which is significantly above the theoretical scale midpoint of 3.0. However, because the PD measure is a non-standard composite and no established clinical or normative thresholds are available, this mean should be interpreted as reflecting moderate levels of distress symptoms in the sample rather than clinically elevated symptomology.

**Table 2:** Descriptive statistics and reliability of study variables (*N* = 689).

Variable	*M*	SD	*α*
Age (years)	20.80	3.20	–
Daily social media use (hrs)	5.30	1.80	–
DSPI	3.03	0.56	0.817
OSC	3.05	0.55	0.777
DIC	3.22	0.54	0.783
FoMO	2.91	0.52	0.716
PD	3.43	0.56	0.841

DSPI, digital self-presentation inauthenticity; OSC, online social comparison; DIC, digital identity confusion; FoMO, fear of missing out, PD, psychological distress, *α*, Cronbach’s alpha. All Likert scales scored 1 to 5.

Mean scores for DIC (*M* = 3.22, *SD* = 0.54), OSC (*M* = 3.05, *SD* = 0.55), and DSPI (*M* = 3.03, *SD* = 0.56) were situated near the midpoint, indicating that these experiences are moderately prevalent in the digital lives of the participants. FoMO yielded the lowest mean score (*M* = 2.91, *SD* = 0.52). Reliability analyses confirmed the psychometric robustness of the instruments, with Cronbach’s alpha (α\alphaα) coefficients ranging from 0.72 to 0.84, indicating acceptable to strong internal consistency across all scales.

### 4.2 Correlation analysis

Pearson correlation coefficients for the primary study variables are presented in Table 3. As hypothesized, DSPI exhibited significant and moderate-to-strong positive correlations with all proposed mediators: OSC (*r* = 0.52, *p* < 0.001), FoMO (*r* = 0.41, *p* < 0.001), and DIC (*r* = 0.40, *p* < 0.001). these results provide preliminary support for H1 and H3.

**Table 3:** Pearson correlation matrix for study variables (*N* = 689).

No.	Variable	M	1	2	3	4	5
1	DSPI	3.03	—				
2	OSC	3.05	0.52^***^	—			
3	DIC	3.22	0.40^***^	0.51^***^	—		
4	FoMO	2.91	0.41^***^	0.35^***^	0.37^***^	—	
5	PD	3.43	0.37^***^	0.37^***^	0.50^***^	0.36^***^	—

DSPI, digital self-presentation inauthenticity; OSC, online social comparison; DIC, digital identity confusion; FoMO, fear of missing out; PD, psychological distress. ^***^p < 0.001.

Furthermore, OSC was strongly associated with DIC (*r* = 0.51, *p* < 0.001), supporting H2. Regarding the primary outcome variable, PD was significantly correlated with all study constructs, showing the strongest associations with DIC (*r* = 0.50, *p* < 0.001) and OSC (*r* = 0.47, *p* < 0.001), followed by DSPI (*r* = 0.37, *p* < 0.001) and FoMO (*r* = 0.36, *p* < 0.001). These findings provide initial empirical support for the pathways predicted in H4. The moderate bivariate relationship between DSPI and PD suggests the presence of significant indirect mechanisms, which are further examined in the subsequent mediation analysis.

### 4.3 Mediation analysis

To test the hypothesized mechanisms linking DSPI to PD, we conducted a combined serial and parallel mediation analysis using the PROCESS macro (Model 81; Hayes, 2018) with 5,000 bootstrap resamples. The results of the path analysis are summarized in Table 1 and Figure 1.

#### 4.3.1 Direct and total effects

The total effect of DSPI on PD (Path c) was statistically significant (*b* = 0.363, SE = 0.035, *p* < 0.001). However, after accounting for the proposed mediators—OSC, DIC, and FoMO—the direct effect (Path c′) was attenuated to non-significance (*b* = 0.071, SE = 0.039, *p* = 0.071). This pattern is consistent with the interpretation that the association between DSPI and PD is statistically accounted for by the variables in the model, with a total indirect effect of *b* = 0.222, SE = 0.029, 95% CI [0.168, 0.283]. Because all variables were measured concurrently, these results represent statistical indirect associations consistent with the hypothesized model rather than evidence of causal mediation.

#### 4.3.2 Indirect pathways

Hypothesis testing of the specific indirect effects confirmed all three proposed pathways.

The Serial Mediation Pathway (H6a): The indirect effect of DSPI on PD through the serial chain of OSC and DIC was significant (*b* = 0.078, SE = 0.018, 95% CI [0.047, 0.116]). Specifically, higher DSPI was associated with increased OSC (*b* = 0.510, *p* < 0.001), which in turn was associated with greater DIC (*b* = 0.399, *p* < 0.001), subsequently was associated with higher PD (*b* = 0.384, *p* < 0.001), consistent with H6a. These associations should be interpreted as concurrent statistical relationships rather than temporal sequences.

The OSC Parallel Pathway (H6b): The parallel indirect effect via OSC alone was also significant (*b* = 0.049, SE = 0.020, 95% CI [0.018, 0.085]), as OSC was significantly associated with distress while controlling for other mediators (*b* = 0.097, *p* = 0.016), consistent with H6b.

The FoMO Parallel Pathway (H6c): Finally, the indirect effect through FoMO was significant (*b* = 0.094, SE = 0.019, 95% CI [0.059, 0.135]). DSPI was a robust statistical predictor of FoMO (*b* = 0.379, *p* < 0.001), which was significantly associated with distress (*b* = 0.247, *p* < 0.001), consistent with H6c.

#### 4.3.3 Comparison of effects

A comparison of the specific indirect effects revealed that the FoMO pathway (*b* = 0.094) yielded the largest magnitude, followed by the serial OSC-DIC chain (*b* = 0.078) and the independent OSC pathway (*b* = 0.049). These findings suggest that, within the concurrent statistical model, the association between DSPI and PD was most strongly accounted for by FoMO, followed by the serial OSC–DIC pathway and the independent OSC pathway. However, because the design is cross-sectional, these patterns represent statistical indirect associations consistent with the hypothesized model and should not be interpreted as evidence of causal mediation.

### 4.4 Multiple regression analysis

To evaluate the unique contributions of the primary study variables and sociodemographic controls (age and gender) to PD, a hierarchical multiple regression analysis was performed. The results are summarized in Table 4.

**Table 4:** Multiple regression: predictors of psychological distress (*N* = 689).

Predictor	*b*	*SE*	*β*	*t*	*p*
(Constant)	1.12	0.21	—	5.33	<0.001
Gender	0.04	0.04	0.03	0.98	0.327
Age	−0.01	0.01	−0.02	−0.54	0.589
DSPI	0.07	0.04	0.07	1.78	0.076
OSC	0.10	0.04	0.10	2.55	0.011
DIC	0.38	0.04	0.37	10.11	<0.001
FoMO	0.25	0.04	0.23	6.68	<0.001

DSPI, digital self-presentation inauthenticity; OSC, online social comparison; DIC, digital identity confusion; FoMO, fear of missing out; PD, psychological distress.

The overall regression model was statistically significant, *F*(6,682) = 56.34, *p* < 0.001, accounting for 33.1% of the total variance in PD (*R^2^* = 0.331, Adj. *R^2^* = 0.326). Among the psychological predictors, DIC emerged as the most robust predictor of distress (*β* = 0.37, *t* = 10.11, *p* < 0.001), followed by FoMO (*β* = 0.23, *t* = 6.68, *p* < 0.001) and OSC (*β* = 0.10, *t* = 2.55, *p* = 0.011).

Consistent with the mediation findings, DSPI was not a significant independent predictor of distress when the mediators were included in the model (*β* = 0.07, *t* = 1.78, *p* = 0.076), indicating that the direct association was no longer statistically significant after accounting for the proposed mediators. This pattern is consistent with indirect associations through the proposed mechanisms but, given the cross-sectional design, should not be interpreted as evidence of full mediation. Furthermore, sociodemographic variables, including age and gender, did not significantly contribute to the variance in PD within this sample (*p* > 0.05), indicating that the observed effects are consistent across different demographic strata.

### 4.5 Exploratory analysis: platform-based differences

To determine whether the primary social media platform utilized by participants influenced their psychological outcomes, a one-way between-subjects analysis of variance (ANOVA) was conducted. Participants were categorized by their primary platform: TikTok (*n* = 215), Instagram (*n* = 198), Twitter/X (*n* = 83), YouTube (*n* = 62), and Multiple Platforms (*n* = 131).

The omnibus test revealed no statistically significant differences in PD across platform groups, *F*(4,684) = 2.23, *p* = 0.064, *η_p_*^2^ = 0.013. Although descriptive trends suggested that primary users of TikTok (*M* = 3.48, *SD* = 0.55) and Instagram (*M* = 3.47, *SD* = 0.57) reported slightly higher levels of distress compared to those using multiple platforms (*M* = 3.32, *SD* = 0.57), these differences did not reach the conventional threshold for statistical significance. These results indicate that no statistically significant differences in psychological distress were detected across platform categories in this sample. However, failure to reject the null hypothesis does not establish equivalence across platforms, and these findings should not be interpreted as evidence that the psychological processes examined here are platform-independent or universal. Demonstrating meaningful equivalence across platforms would require a different analytical framework.

## 5 Discussion

The present study investigated the psychological mechanisms linking DSPI to PD among Malaysian youth. By integrating Goffman’s (1959) dramaturgical framework with contemporary digital affordance theories (Soh et al., 2024; Valkenburg et al., 2022), we identified three distinct mediated pathways: a serial chain through OSC and DIC, and two parallel pathways through OSC and FoMO. Notably, the direct association between DSPI and PD was no longer statistically significant after accounting for the proposed mediators. However, because the design was cross-sectional, this pattern should not be interpreted as evidence of full mediation or causal transmission; the concurrent measurement of all variables precludes causal or temporal inference.

H1, which proposed that DSPI is positively associated with OSC, was supported. This finding aligns with prior research indicating that individuals who perceive their online self-presentation as inauthentic become more attentive to how their presentation compares with those of others (Bailey et al., 2020; Türk-Kurtça and Doğru, 2026). Türk-Kurtça and Doğru (2026) similarly found that digital esthetics influenced social media self-presentation through the serial mediation of social media comparison and false self-presentation. A possible explanation is that perceived inauthenticity heightens self-evaluative concerns, prompting individuals to seek external referents for evaluating their own online identity, consistent with Festinger’s (1954) SCT. This finding extends prior work by demonstrating that DSPI, specifically, is associated with OSC, rather than merely general social-media use.

H2, which proposed that OSC is positively associated with DIC, was supported. This finding is consistent with prior research linking social-media comparison to lower identity clarity and greater identity distress (Yang et al., 2018; Zhao and Li, 2026). Zhao and Li (2026) demonstrated that self-discrepancy and ideal internalization function as key mediators linking digital social comparison to impaired self-concept over time. Repeated exposure to curated representations of others may draw attention to discrepancies between how individuals understand themselves and how they believe they should present themselves online, making it more difficult to maintain a coherent identity across contexts (Higgins, 1987). This finding is notable because it links OSC specifically to DIC, a construct that captures identity confusion arising from digital engagement, rather than to broader identity distress.

H3, which proposed that DSPI is positively associated with FoMO, was supported. This finding extends prior research on FoMO (Przybylski et al., 2013) by identifying DSPI as a correlate of FoMO. Individuals who perceive their digital self-presentation as inauthentic may become more attentive to socially desirable experiences displayed by others, heightening concern about whether their own experiences are sufficiently socially rewarding. From a dramaturgical perspective (Goffman, 1959), individuals managing their own digital performances are simultaneously exposed to the performances of others, which may intensify concerns about social exclusion and missed experiences. This finding contributes to the literature by showing that DSPI is not only associated with social comparison but also with affective concerns about missing out.

H4, which proposed that DIC is positively associated with PD, was supported. In the regression analysis, DIC emerged as the strongest proximal predictor of distress. This finding is consistent with prior research linking identity confusion to less truthful and more socially desirable self-presentation (Michikyan, 2020) and with evidence that self-concept clarity is associated with psychological functioning (Campbell et al., 1996). Persistent uncertainty about the coherence or continuity of the self may be psychologically burdensome, particularly during adolescence and emerging adulthood when identity development remains important (Higgins, 1987). This finding is particularly significant because it identifies DIC as the strongest proximal predictor of distress among the mediators examined, highlighting the centrality of identity processes in digital mental health. H5, which proposed that FoMO is positively associated with PD, was supported. This finding is consistent with prior research linking FoMO with poorer mood, lower life satisfaction, and greater social-media engagement (Przybylski et al., 2013), as well as with large-scale evidence that FoMO operates as a distinct pathway to psychological distress (Ho et al., 2026). Cahyaningthyas and Huwae (2026) similarly reported a significant positive correlation between FoMO and psychological distress. Individuals experiencing FoMO may have persistent concerns about social exclusion, disconnection, and inadequate participation, which may be reinforced by continuous access to social information. This finding corroborates and extends prior research by demonstrating that FoMO is associated with distress even when controlling for other mediators.

H6a, which proposed that DSPI has a positive indirect association with PD through the serial pathway of OSC and DIC, was supported. This finding is consistent with prior research demonstrating that social media comparison and false self-presentation sequentially mediate the relationship between digital esthetics and social-media self-presentation outcomes (Türk-Kurtça and Doğru, 2026) and that self-discrepancy and ideal internalization mediate the longitudinal effects of digital social comparison on self-concept (Zhao and Li, 2026). This finding provides a mechanistic account of how DSPI may be associated with PD through a sequential process involving both comparative evaluation and identity confusion. H6b, which proposed that DSPI has a positive indirect association with PD through OSC, was supported. This finding is consistent with prior research linking online social comparison to adverse self-evaluative outcomes, including body-image concerns (Bonfanti et al., 2025) and sustained negative effects on self-concept (Zhao and Li, 2026). This finding suggests that OSC may operate as a distinct pathway to distress, independent of identity confusion. H6c, which proposed that DSPI has a positive indirect association with PD through FoMO, was supported. Notably, the FoMO pathway yielded the largest magnitude among the specific indirect effects. This finding extends prior research by identifying FoMO as a distinct pathway to distress (Ho et al., 2026; Cahyaningthyas and Huwae, 2026). Perceived DSPI may be associated with greater sensitivity to the social experiences displayed by others, while continuous exposure to such experiences may correspond with concerns about exclusion and social disconnection (Przybylski et al., 2013). This finding is particularly noteworthy because it suggests that FoMO may be a more potent indirect pathway than OSC in the context of DSPI.

The findings are broadly consistent with prior research on digital self-presentation, social comparison, identity confusion, FoMO, and psychological distress. However, some differences warrant consideration. First, while prior research has often focused on single mechanisms (e.g., social comparison or FoMO), the present study examined multiple mechanisms concurrently within a single model. This approach revealed that FoMO was the most potent indirect pathway, whereas prior research has often emphasized social comparison as the primary driver of digital malaise (Bonfanti et al., 2025). One possible explanation is that the present study was conducted in a Malaysian context, where collectivist cultural values may heighten concerns about social exclusion and belongingness. This cultural context may amplify the salience of FoMO relative to OSC, a possibility that warrants further cross-cultural investigation. Second, while prior research has linked social-media use to diminished wellbeing (Hancock et al., 2022), the present study found that DSPI, rather than usage frequency or platform type, was more consistently associated with distress. This finding supports calls for more nuanced, person-centered approaches in media effects research (Valkenburg et al., 2022). It also suggests that the qualitative nature of digital engagement, rather than the quantity or platform of use, may be more consequential for psychological adjustment.

## 6 Implications

This study advances digital psychology in four key areas. First, it provides a rare empirical test of a combined serial and parallel mediation model, moving beyond single-mechanism accounts to capture the complexity of digital life. Second, it provides initial evidence for DIC as a distinct, measurable construct tailored for the social media era, filling a gap in traditional identity research. However, further validation in independent samples remains warranted. Third, it challenges the dominance of “social comparison” narratives by highlighting FoMO as the more powerful affective driver of distress in youth. Finally, it establishes an authenticity-centered framework for mental health, suggesting that future research should focus on the psychological “how” of social media use rather than the technological “where.” The findings of this study offer preliminary directions for future research and intervention development. Because the design was cross-sectional, these implications should be interpreted as hypothesis-generating rather than as empirically supported recommendations for clinical practice or public health policy.

The non-significant relationship between platform type and PD suggests that future research should examine the qualitative nature of engagement specifically, the cultivation of digital authenticity rather than focusing exclusively on platform-specific effects. However, because a non-significant omnibus test does not establish equivalence, these findings should not be interpreted as evidence that the processes examined here are platform-independent. Future research using equivalence testing or larger platform-specific samples is needed to examine this question.

A notable finding of this research is the identification of DIC as the strongest proximal predictor of PD. This finding suggests that future research should examine whether assessments of digital identity may be useful in understanding psychological distress. However, because the present study did not evaluate a diagnostic instrument or an intervention, recommendations for clinical practice would be premature. Future research should first develop and validate measures of digital identity confusion before considering their potential clinical utility. Mental health practitioners may consider exploring digital identity concerns with young clients, but such assessments should be viewed as exploratory rather than diagnostic. Educational curricula may benefit from teaching the psychological mechanisms of the “digital performance cycle.” By explaining how inauthenticity may be associated with social comparison and FoMO, schools may help youth recognize these triggers in real-time. However, because the present study did not evaluate an educational intervention, these suggestions should be viewed as directions for future intervention development rather than as empirically supported recommendations. Programs that encourage youth to bridge the “authenticity gap” by posting content that reflects lived experiences rather than idealized performances may simultaneously mitigate the multiple indirect pathways to distress identified in our model, but their effectiveness should be evaluated through longitudinal or experimental research. In summary, the findings of this study provide a foundation for future research on digital authenticity and mental health. However, because the design was cross-sectional, these results should be used to inform hypothesis generation and intervention targets rather than to establish causal pathways or to support immediate changes in clinical practice or public health policy.

## 7 Limitations and directions for future research

Several limitations may have influenced the results. First, the cross-sectional nature of the data precludes definitive causal inferences. While the proposed pathways are theoretically grounded in dramaturgical and self-discrepancy frameworks, the observed relationships may be bidirectional. For instance, individuals experiencing higher levels of baseline PD might engage in inauthentic self-presentation as a maladaptive coping mechanism to bolster perceived social capital. Future research should employ longitudinal designs or experience sampling methodology (ESM) to capture within-person fluctuations in digital performance and wellbeing. Adopting person-specific paradigms, such as N-of-1 dynamic structural equation modeling (Valkenburg et al., 2022), would further clarify the temporal precedence of these effects. The observed indirect associations are consistent with the hypothesized model but do not establish that one psychological process temporally produces another.

Second, the study relied exclusively on self-report measures, which may introduce common-method bias and social desirability effects, particularly regarding sensitive topics like inauthenticity and distress. Future studies should integrate multi-method approaches, such as objective coding of social media content (e.g., analyzing image idealization) or digital footprint data (e.g., editing time and filter usage). Experimental designs that manipulate self-presentation conditions (authentic vs. inauthentic) would also provide a more robust test of the causal links hypothesized here. Third, while the DIC scale demonstrated strong internal consistency, it was developed specifically for this study and has not undergone extensive psychometric validation. Prioritizing confirmatory factor analysis (CFA) in independent samples and testing for convergent validity against established self-concept clarity measures are essential next steps for the field.

Fourth, although the sample was broadly comparable to the Malaysian national census in ethnic composition, the use of purposive and snowball sampling means that the sample cannot be considered nationally representative. The similarity with census proportions is reported descriptively to indicate ethnic diversity and does not establish population-level generalizability. Additionally, the focus on youth aged 15–30 may limit generalizability to other developmental stages. The mechanisms of identity confusion may be more pronounced in early adolescence—a critical period for identity formation—compared to older adults with more consolidated self-concepts. Furthermore, cross-cultural research is necessary to determine if these pathways are influenced by Malaysia’s specific collectivist orientation or if they represent universal digital dynamics.

Finally, this study did not account for potential buffering factors. Future research should explore moderators such as self-compassion, digital literacy, and offline social support, which may mitigate the deleterious effects of inauthentic performance. Additionally, while the non-significant platform differences suggest a person-specific effect, future research with larger, platform-stratified samples may reveal more subtle affordance-based variations that the current study was not powered to detect. However, because a non-significant omnibus test does not establish equivalence, these findings should not be interpreted as evidence that the processes examined here are platform-independent.

## 8 Conclusion

As digital environments have become a primary stage for identity development, the discrepancy between the lived self and the performed self has emerged as a significant determinant of mental health. This study, involving 689 Malaysian youth, provides empirical evidence that DSPI is statistically associated with a complex cascade of PD through a combined serial and parallel mediation framework, with statistical associations consistent with transmission through three distinct mechanisms: a serial path via OSC and DIC, and two parallel paths via OSC and FoMO. These findings offer evidence of statistical indirect associations between DSPI and PD through OSC, DIC, and FoMO, and although consistent with the hypothesized model, they should not be interpreted as evidence of temporal or causal mediation given the cross-sectional design; future research using longitudinal or experimental designs is needed to examine directionality. The findings reveal a critical hierarchy of digital harms, with FoMO representing the most potent indirect pathway—capturing the pervasive anxiety of social exclusion—while DIC emerged as the strongest proximal predictor of distress, suggesting that fragmentation of the self-concept is the most debilitating consequence of the “authenticity gap.” No statistically significant differences in PD were detected across platform categories, though this does not establish equivalence across platforms, and further research is needed to examine platform-specific processes. Ultimately, the curation of an idealized digital persona is far from a benign esthetic choice; it is a psychologically consequential act concurrently associated with destabilized wellbeing, and by mapping the pathways from inauthenticity to distress, this study provides a theoretical and practical foundation for moving beyond “screen time” debates toward a more nuanced, authenticity-centered approach to digital mental health. However, because the design was cross-sectional, these findings should be used to inform hypothesis generation and intervention targets rather than to establish causal pathways.

## Data Availability

The raw data supporting the conclusions of this article will be made available by the authors, without undue reservation.
